# In Vivo Assessment of Exercise-Induced Glenohumeral Cartilage Strain

**DOI:** 10.1177/2325967118784518

**Published:** 2018-07-13

**Authors:** Hanci Zhang, Lauren N. Heckelman, Charles E. Spritzer, Kwadwo A. Owusu-Akyaw, John T. Martin, Dean C. Taylor, C.T. Moorman, Grant E. Garrigues, Louis E. DeFrate

**Affiliations:** * Department of Orthopaedic Surgery, James R. Urbaniak, MD, Sports Sciences Institute, Duke University, Durham, North Carolina, USA.; † Department of Biomedical Engineering, Duke University, Durham, North Carolina, USA.; ‡ Department of Radiology, Duke University, Durham, North Carolina, USA.; § Department of Mechanical Engineering and Materials Science, Duke University, Durham, North Carolina, USA.; *Investigation performed at Duke University, Durham, North Carolina, USA*

**Keywords:** articular cartilage, biomechanics, magnetic resonance imaging (MRI), shoulder

## Abstract

**Background:**

The human shoulder joint is the most mobile joint in the body. While in vivo shoulder kinematics under minimally loaded conditions have been studied, it is unclear how glenohumeral cartilage responds to high-demand loaded exercise.

**Hypothesis:**

A high-demand upper extremity exercise, push-ups, will induce compressive strain in the glenohumeral articular cartilage, which can be measured with validated magnetic resonance imaging (MRI)–based techniques.

**Study Design:**

Descriptive laboratory study.

**Methods:**

High-resolution MRI was used to measure in vivo glenohumeral cartilage thickness before and after exercise among 8 study participants with no history of upper extremity injury or disease. Manual MRI segmentation and 3-dimensional modeling techniques were used to generate pre- and postexercise thickness maps of the humeral head and glenoid cartilage. Strain was calculated as the difference between pre- and postexercise cartilage thickness, normalized to the pre-exercise cartilage thickness.

**Results:**

Significant compressive cartilage strains of 17% ± 6% and 15% ± 7% (mean ± 95% CI) were detected in the humeral head and glenoid cartilage, respectively. The anterior region of the glenoid cartilage experienced a significantly higher mean strain (19% ± 6%) than the posterior region of the glenoid cartilage (12% ± 8%). No significant regional differences in postexercise humeral head cartilage strain were observed.

**Conclusion:**

Push-ups induce compressive strain on the glenohumeral joint articular cartilage, particularly at the anterior glenoid. This MRI-based methodology can be applied to further the understanding of chondral changes in the shoulder under high-demand loading conditions.

**Clinical Relevance:**

These results improve the understanding of healthy glenohumeral cartilage mechanics in response to loaded upper extremity exercise. In the future, these methods can be applied to identify which activities induce high glenohumeral cartilage strains and deviations from normal shoulder function.

The shoulder joint, composed of the proximal humerus, scapula, and clavicle, is the most mobile joint in the human body.^27^ This mobility is important for performing many physiologic functions, including sports and exercise. While in vivo shoulder joint kinematics have been studied through various noninvasive imaging techniques, they have been examined primarily under minimally loaded or unloaded conditions. For example, a previous study demonstrated anteroposterior translation of the humeral head during external and internal rotation with biplanar fluoroscopy.^4^ Other specific motions studied in the literature include coronal plane abduction and scapular elevation.^2–5,18,19,26^ While these studies are important foundations for understanding glenohumeral joint mechanics, glenohumeral kinematics during loading consistent with physical exercise and sports remain incompletely described in vivo.

During exercise, the shoulder musculature contracts in a coordinated fashion to maintain joint stability throughout a full range of motion.^17^ Dynamic muscular activity is the primary stabilizer of the glenohumeral joint, which is in contrast to joints such as the knee or ankle, where ligamentous structures or bony architecture play important roles in stability.^17,25^ As such, the glenohumeral joint is subject to large joint forces generated by muscular contraction. The magnitudes of these forces, even during routine activities of daily living, can exceed total body weight.^33^ During more intense upper extremity activities, these forces are likely much greater still, but it is unknown how glenohumeral cartilage is influenced by or responds to these stresses. Furthermore, models of glenohumeral contact patterns constructed with cartilage surface data differ significantly from models constructed with only the subchondral bone surface,^5,19^ indicating that the interaction between the glenoid and humeral head articular surfaces must be taken into account to accurately assess shoulder cartilage loading.

Although direct measurements of localized glenohumeral cartilage deformations in response to dynamic joint loading are lacking, our laboratory previously investigated exercise-induced tibiofemoral and tibiotalar cartilage deformations in vivo with magnetic resonance imaging (MRI) and 3-dimensional (3D) modeling.^8,15,16,29^ This technique takes advantage of cartilage’s biphasic nature.^20,21^ Fluid is exuded from the extracellular matrix of the cartilage when subjected to a mechanical load, and upon removal of the load, fluid re-enters the extracellular matrix in a time-dependent manner.^20,21^ This time dependency enables the use of MRI to quantify cartilage deformations immediately postexercise, before the cartilage has had time to fully recover to its baseline thickness.

The aim of this study was to use these previously validated MRI-based 3D solid modeling techniques^8,9,15,16,29,30^ to evaluate site-specific glenohumeral cartilage deformations in response to a series of push-ups. We hypothesized that a series of 30 push-ups would result in measurable nonuniform glenohumeral cartilage deformations across the humeral head and glenoid, as quantified with MRI-based 3D solid modeling techniques.

## Methods

### Participant Recruitment

Ten healthy participants (7 men, 3 women; mean age, 26 years [range, 22-28 years]; mean body mass index [BMI], 22.9 kg/m^2^ [range, 20.0-30.2 kg/m^2^]) were recruited to undergo exercise testing in this institutional review board–approved study. Exclusion criteria included any history of shoulder-related injuries, symptoms, treatments, or surgery. During the study, 2 men were found to have asymptomatic defects in the glenoid labrum or cartilage and were excluded from the analysis. Therefore, data from 5 men and 3 women were included in the following analyses.

### Imaging and Exercise Protocol

Data collection for all participants began at 7 am to reduce the potential for diurnal changes in cartilage thickness.^9,34^ Additionally, participants were asked to refrain from any strenuous upper body exercise or weight lifting in the 24 hours prior, and they rested supine for at least 45 minutes to allow for cartilage equilibration prior to pre-exercise MRI.^9,29^ Then, pre-exercise magnetic resonance (MR) images of each participant’s dominant shoulder (2 left, 6 right) were obtained with a 3.0-T MR scanner (Trio Tim; Siemens), a dedicated shoulder coil, and a balanced steady-state gradient-echo pulse sequence (TruFISP; flip angle: 20°/40°, repetition time: 8.9 milliseconds, echo time: 3.9 milliseconds, orientation: axial, resolution: 0.3 × 0.3 × 0.5 mm^3^, field of view: 160 × 160 mm, matrix size: 512 × 512 pixels, slice thickness: 0.5 mm).

Participants then performed a series of 30 standardized push-ups, keeping their palms shoulder-width apart and their elbows close to their body to avoid shoulder abduction throughout the exercise. All participants were supervised by a research team member to ensure adherence to the correct form, and all participants completed the full series of push-ups within 100 seconds. Immediately after exercise, participants were transported back to the MR scanner for imaging per the parameters outlined earlier. TruFISP image acquisition began within 3 minutes after exercise conclusion. The total scan time for each TruFISP sequence was 6 minutes 31 seconds, taken pre- and postexercise.

### Image Analysis

**Figure 1.**
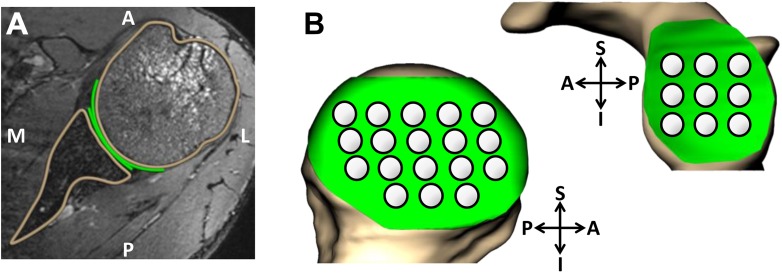
(A) Axial magnetic resonance image of a left shoulder. (B) Humeral head and glenoid articular cartilage grid systems for measuring regional changes in thickness. A, anterior; I, inferior; L, lateral; M, medial; P, posterior; S, superior.

The pre- and postexercise MR images were imported into solid modeling software (Rhinoceros; Robert McNeel and Associates) to generate 3D models of the humerus, scapula, and glenohumeral cartilage. A single investigator (H.Z.) manually segmented the bony cortices and cartilage surfaces of the humeral head and glenoid. The same investigator also traced 10 MR slices from a single participant a total of 4 times each, providing data from which to compute the repeatability of the manual segmentation process. The mean glenohumeral cartilage thickness across all trials was repeatable to within a standard deviation of 0.04 mm, corresponding to a cartilage strain of approximately 3.5%. A subpixel level of precision was made possible by segmenting the images with NURBS (nonuniform rational b-splines), which bisect the rectilinear image matrix. Specifically, this technique leverages signal intensity gradients across multiple pixels in the images to precisely identify the bone and cartilage surfaces during the manual segmentation process. This method was previously validated and has been extensively used to quantify cartilage thickness in vivo in other joints.^8,9,15,16,24,29,32^ Additionally, to ensure quality control, all segmentations were reviewed by a fellowship-trained musculoskeletal radiologist with 30 years of experience (C.E.S.). The bone and cartilage contours were then compiled to create a 3D surface mesh model of the humerus, the glenoid, and the respective cartilage surfaces (Geomagic Studio; Geomagic) (Figure 1A).

Pre- and postexercise bony surfaces were aligned with an iterative closest-point technique, enabling a site-specific comparison of cartilage thickness before and after exercise. Cartilage thickness maps were generated by finding the distance between each vertex on the bone surfaces and the nearest vertex on the corresponding cartilage surfaces. Localized thickness measurements were computed within 2-mm radius sampling regions spanning the cartilage surface (18 on the humeral head and 9 on the glenoid) (Figure 1B). Strain within each sampling region was defined as the change in cartilage thickness postexercise relative to pre-exercise, normalized to the pre-exercise cartilage thickness. Overall compartmental strains were defined as the mean strain across all sampling regions on the glenoid and humeral head cartilage surfaces, respectively.

### Statistical Analysis

Routine descriptive statistics were used to summarize the data. Repeated-measures analyses of variance (ANOVAs) tested for overall and localized differences in humeral head and glenoid cartilage thickness pre- and postexercise. Significant results were followed up with Tukey post hoc tests. Two separate repeated-measures ANOVAs were used to compare regional glenoid cartilage strains along the superoinferior (superior, central, and inferior) and anteroposterior (anterior, central, and posterior) axes. Furthermore, a final repeated-measures ANOVA compared regional humeral head cartilage strains between the superior and inferior regions, as well as along the anteroposterior axis (anterior, central, and posterior) of the humeral head. Pearson correlations were computed to assess the relationships between BMI and body weight on the observed humeral head and glenoid cartilage strains. All statistical analyses were performed with Statistica (StatSoft). Statistical significance was established where *P* < .05. All results are presented as mean ± 95% CI.

## Results

**Figure 2.**
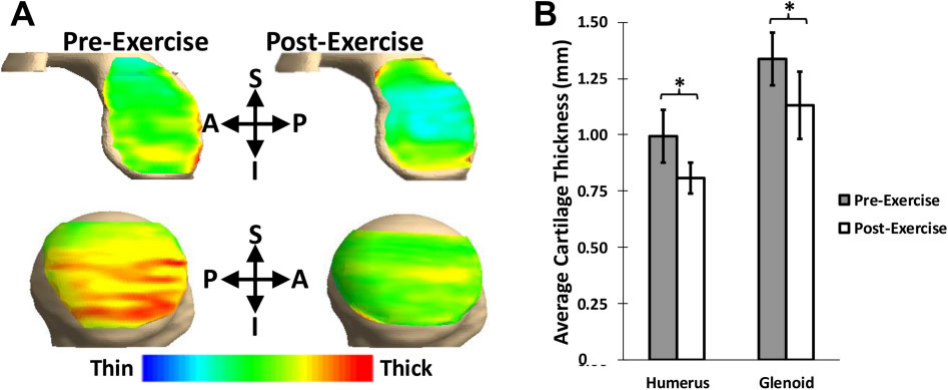
(A) Glenoid cartilage thickness maps. Blue represents the thinnest cartilage, while red represents the thickest cartilage. (B) Significant cartilage thickness changes (mean ± 95% CI) were observed at the humerus (*P = .002) and the glenoid (*P = .003) immediately after push-ups. A, anterior; I, inferior; P, posterior; S, superior.

Pre-exercise cartilage thicknesses were 1.0 ± 0.1 mm on the humeral head and 1.3 ± 0.1 mm on the glenoid (Figure 2). We did not detect significant site-specific differences in baseline humeral head cartilage thickness (*P* = .777); however, site-specific differences in baseline glenoid cartilage thickness were observed (*P* < .001). Specifically, pre-exercise glenoid cartilage was thinnest in the center (1.2 ± 0.2 mm) and thicker in the peripheral areas, with the thickest cartilage present in the anteroinferior region (1.5 ± 0.2 mm). Following activity, the mean cartilage thicknesses significantly decreased to 0.8 ± 0.1 mm on the humeral head (*P* = .002) and 1.1 ± 0.2 mm on the glenoid (*P* = .003) (Figure 2). There were no site-specific differences seen in postexercise humeral head (*P* = .916) or glenoid cartilage (*P* = .096) thicknesses.

**Figure 3.**
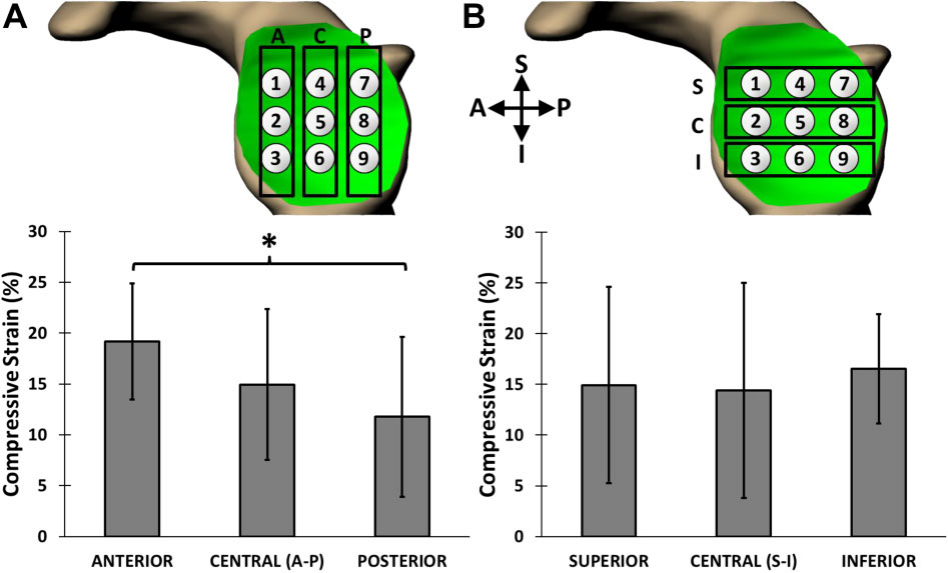
Regional postexercise strains (mean ± 95% CI) at the glenoid. (A) Anteroposterior axis. Higher strain was observed in the anterior region of the glenoid cartilage as compared with the posterior region (*P = .005). (B) Superoinferior axis. No regional differences in strain were observed (*P* = .992). A, anterior; C, central; I, inferior; P, posterior; S, superior.

**Figure 4.**
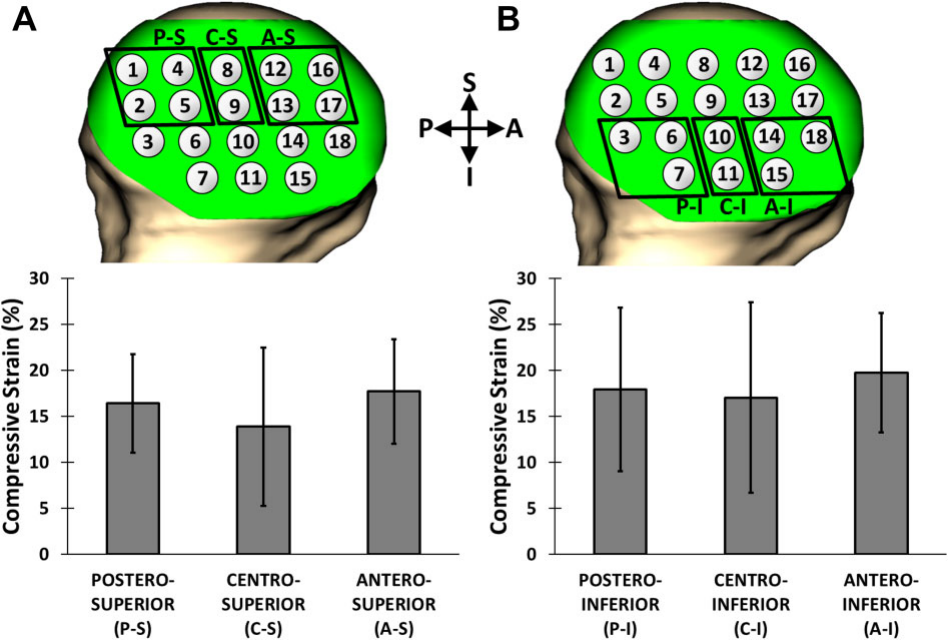
Regional postexercise strains (mean ± 95% CI) at the humeral head. Anteroposterior axis on the (A) superior humeral head and (B) inferior humeral head. No regional differences in cartilage strain were observed (*P* = .363). A, anterior; C, central; I, inferior; P, posterior; S, superior.

Significant compressive strains were detected in the humeral head and glenoid cartilage after the series of 30 push-ups. The overall compressive strain was 17% ± 6% across the humeral head cartilage and 15% ± 7% across the glenoid cartilage. BMI and body weight were not shown to be correlated with humeral head (*P* = .443 and .515) or glenoid (*P* = .687 and .460) cartilage strain. The mean compressive strain in the anterior glenoid cartilage (19% ± 6%) was significantly greater than that in the posterior glenoid cartilage (12% ± 8%, *P* = .005), but neither region was shown to be significantly different from the central region of the glenoid cartilage (15% ± 7%) (Figure 3). In contrast, we did not detect significant differences in strain along the superoinferior axis of the glenoid (*P* = .992). There were no regional differences in strain observed along the anteroposterior (*P* = .363) or superoinferior (*P* = .363) axes of the humeral head (Figure 4). Local strain maxima were observed at anteroinferior (Figure 4, points 14, 15, and 18) (range, 17%-22%) and posteroinferior (Figure 4, points 3, 6, and 7) (range, 14%-20%) locations on the humeral head. Local strain minima were seen in the superocentral region of the humeral head (Figure 4, points 8 and 9) (range, 13%-15%).

## Discussion

Physiologic glenohumeral cartilage function in response to loading has not been fully characterized in vivo. This investigation quantified glenohumeral cartilage deformations in response to loaded exercise with previously validated MRI-based 3D solid modeling techniques.^8,9,15,16,24,29,32^ Specifically, this study evaluated exercise-induced glenohumeral cartilage strain following a series of 30 push-ups. Compressive strains were measured in the glenoid (15%) and humeral head (17%) articular cartilage. Regional differences in strain were observed along the anteroposterior axis of the glenoid, with a significantly higher strain measured in the anterior third (19%) of the glenoid cartilage as compared with the posterior third (12%). However, no regional differences in strain were observed along the superoinferior axis of the glenoid or the anteroposterior and superoinferior axes of the humeral head.

The glenoid and humeral head cartilages responded differently to the push-up sequence. Glenoid cartilage strain was nonuniform, as hypothesized, as the anteroinferior region of the glenoid cartilage experienced the greatest strain. Near the highest point of the push-up cycle, scapular protraction and upward rotation place the glenoid parallel to the floor, and the humerus is relatively neutral in rotation. In contrast, near the lowest point of the cycle, the glenoid is perpendicular to the floor, owing to scapular retraction and downward rotation. Additionally, the humerus externally rotates near the lowest point of the push-up cycle, which translates the humeral head anteriorly within the glenoid.^4^ This anterior translation may explain why the highest strains were measured in the anterior region of the glenoid cartilage. Congruently, previous work by Massimini et al^19^ linked humeral external rotation during scapular elevation-depression with anteroinferior glenohumeral cartilage contact patterns. Conversely, no significant regional variations in strain were detected across the humeral head cartilage. The observed results may be due to the larger surface area of the humeral head relative to the glenoid.^14,28^ Specifically, as the humeral head rotates within the glenoid as the arm moves, different areas of the humeral head may be in contact with the glenoid. This varying contact region may help to more evenly distribute strain across the humeral head.

In this study, overall glenohumeral cartilage strains were notably greater than those previously measured in the knee and ankle following a weightbearing impact exercise (a series of single-legged hops) in work by members of our research group.^8,29^ For instance, Sutter et al^29^ observed overall compressive cartilage strains of 5% at the tibia, with smaller strains at the femur (1%-2%). Furthermore, when investigating ankle cartilage deformations in response to single-legged hops, Cher et al^8^ observed overall compressive strains of 3% at the tibia and 2% at the talus. Variations in cartilage loading patterns associated with cartilage-specific material properties,^31^ different exercise protocols, and anatomic functions may account for the differences in strain magnitudes measured in the current shoulder study (12%-19%) as compared with those previously measured in cartilage of the lower extremity. Ankle cartilage, for instance, has a higher aggregate (equilibrium) modulus, higher dynamic stiffness, and lower water permeability than knee cartilage.^31^

Future investigations are required to discern whether the physical properties of glenohumeral cartilage differ from those of cartilage within lower extremity joints. This may be explored with quantitative MRI techniques, such as T2, T2*, and T1rho mapping, which has been correlated with the structure and composition of cartilage.^6,10,12,13,23,35^ Specifically, T2, T2*, and T1rho relaxation times are associated with collagen alignment and proteoglycan concentration, which are linked to tissue material properties.^6,13,35^ Additionally, although the shoulder is not generally considered a weightbearing joint, intra-articular forces within the glenohumeral joint eclipsing body weight may be possible even during routine activities of daily living.^33^ By extension, increased rotator cuff activation during sports and exercise is likely to result in even greater forces in the joint, which may lead to higher compressive strains in the tissue. To this point, previous investigations used motion capture and inverse dynamics techniques to assess intra-articular forces in the glenohumeral joint.^1,22^ In the future, our MRI-based technique can be used to provide boundary conditions for computational shoulder models to estimate intra-articular forces from the strain distributions in the joint.

Furthermore, differences in anatomic function may drive differences in cartilage response to loading. The tibiofemoral and tibiotalar joints are repeatedly subjected to full body weight loading during activities of daily living, including during typical bipedal walking and standing. As a result, these joints may be better adapted to handle the high stresses incurred during exercise, resulting in a lower magnitude of compressive strain. In comparison, the glenohumeral joint may be more susceptible to cartilage strain when placed under weightbearing loads (ie, push-ups) as compared with the knee and ankle joints.

The MRI-based technique chosen for this study quantified the cumulative effect of push-ups on glenohumeral cartilage by measuring strain immediately postexercise, providing insight into the loading experienced during strenuous exercise. Future studies pairing MRI thickness measurements of cartilage with other techniques, such as biplanar radiography, may provide additional information regarding time-dependent cartilage deformations during exercise.^7^ Note that, based on cartilage recovery, instantaneous cartilage strains during push-ups may be even greater than the strains observed after exercise in this study. The protocol and experimental setup were designed to minimize the time between the last recorded push-up and the completion of MRI, as this would decrease the effect of cartilage recovery. A previous investigation in patellar cartilage showed that the cartilage can recover to approximately 50% of its baseline thickness within 45 minutes after a series of 100 knee bends.^11^ While the time between the final push-up and the start of anatomic MR image acquisition in this study was much less than that time frame (<3 minutes), specific glenohumeral cartilage recovery times are currently unknown. Quantifying glenohumeral cartilage recovery timelines in vivo is a goal for future investigations.

This study demonstrated how MRI-based measurement techniques can be used to evaluate glenohumeral cartilage deformations in vivo, which is an important step toward understanding shoulder cartilage mechanics, not only during isolated motions but also during high-demand sports and exercise. In particular, a series of just 30 push-ups induced significant compressive glenohumeral cartilage strain. Push-ups were selected for this investigation because they are a common upper extremity exercise familiar to the general population. Although this study investigated a series of 30 push-ups specifically, this methodology may also be used to test how dosage (ie, the number of push-ups performed) relates to glenohumeral cartilage strain magnitudes. This technique can also be applied to a range of other upper extremity exercises, which could be used to investigate how these activities affect glenohumeral cartilage deformations. Finally, this technique can be used to investigate whether cartilage strain distributions change as a result of labral or rotator cuff instability or tears, and it can be implemented to determine if surgical interventions can restore the deformation patterns in the cartilage. Thus, the results of this investigation complement the existing understanding of shoulder kinematics during range of motion and minimally loaded exercises and potentially have implications for exercise science and the prevention of athletic injury.

## Conclusion

Push-ups induce significant compressive glenohumeral cartilage strain that can be reliably measured noninvasively in vivo with MRI techniques. Although the present study represents a first description of this technique to measure cartilage strain in the shoulder, it serves as a foundation for future investigations on how glenohumeral cartilage is affected by loading, exercise, and injury.
